# Medical emergencies on board commercial airlines: is documentation as expected?

**DOI:** 10.1186/cc11238

**Published:** 2012-03-07

**Authors:** Michael Sand, Stephan Morrosch, Daniel Sand, Peter Altmeyer, Falk G Bechara

**Affiliations:** 1 Department of Dermatology, Venereology and Allergology, Ruhr-University Bochum, Gudrunstr. 56, 44791 Bochum, Germany; 2 Department of Anaesthesiology and Intensive Care Medicine, St. Josef Hospital, Ruhr-University Bochum, Gudrunstr. 56, 44791 Bochum, Germany; 3 Department of Medicine, Olive View UCLA Medical Center, University of California, Los Angeles (UCLA), 14445 Olive View Drive, Sylmar, CA 91342, USA

## Abstract

**Introduction:**

The purpose of this study was to perform a descriptive, content-based analysis on the different forms of documentation for in-flight medical emergencies that are currently provided in the emergency medical kits on board commercial airlines.

**Methods:**

Passenger airlines in the World Airline Directory were contacted between March and May 2011. For each participating airline, sample in-flight medical emergency documentation forms were obtained. All items in the sample documentation forms were subjected to a descriptive analysis and compared to a sample "medical incident report" form published by the International Air Transport Association (IATA).

**Results:**

A total of 1,318 airlines were contacted. Ten airlines agreed to participate in the study and provided a copy of their documentation forms. A descriptive analysis revealed a total of 199 different items, which were summarized into five sub-categories: non-medical data (63), signs and symptoms (68), diagnosis (26), treatment (22) and outcome (20).

**Conclusions:**

The data in this study illustrate a large variation in the documentation of in-flight medical emergencies by different airlines. A higher degree of standardization is preferable to increase the data quality in epidemiologic aeromedical research in the future.

## Introduction

Air travel has emerged as one of the most popular, safe and convenient forms of travel. In the past decade, the number of passengers travelling on commercial airlines has increased to almost two billion [1]. Because we live in an aging society, the average age of the passengers who are travelling with chronic disease and the number of chronic diseases per passenger is likely to increase in the future. For the European Union (EU), the EU's statistical analysis unit, Eurostat, has calculated that in the year 2060 over 30% of the entire EU population will be over the age of 65. For 2008, Eurostat reported the population over the age of 65 to be 17.1%. However, it is important to keep in mind that these statistics are solely based on age and have no relation to the actual health status of the EU population. Although it is likely that increasing age goes along with chronic diseases, it is not possible to make definite conclusions regarding the health status of future travelers around the world based on EU data [2]. Nevertheless, despite the fact that air travel is generally safe, an increase in in-flight medical emergencies is expected [3]. The next generation of aircrafts, such as the Airbus A380-900 (Airbus S.A.S., Toulouse, France) and the Boeing 777 LR (Boeing Commercial Airplanes, Renton, WA, USA), have an estimated cruising range of 15 to 17,000 km and a maximum passenger load of up to 960 passengers, which will further increase the chances that an in-flight medical emergency will occur during each flight [3].

A recent study reported preliminary evidence that the documentation of in-flight medical emergencies is not as consistent as one would expect. Of the 32 European airlines that were asked to contribute data on in-flight medical emergencies, only four airlines were able to potentially provide the necessary data [4]. In a commentary on the latter study, Ruskin discussed the idea of establishing an international registry of in-flight medical emergencies [5]. Thus, the present study was initiated as a descriptive baseline study to describe the documentation forms that are currently in use. Additionally, the data were compared to the recommendations of the International Air Transport Association (IATA), which is the largest airline-representing association worldwide and which has published a sample "medical incident form" in their Medical Manual [6].

## Materials and methods

This study conforms to the applicable local requirements regarding the ethical and investigational committee review, informed consent, and other statutes or regulations regarding the protection of the rights and welfare of the human subjects participating in medical research (http://ClinicalTrials.gov Identifier: NCT01477684, approved by the Ethical Review Board of the Ruhr-University Bochum, Germany, registration number: 4096-11). This study originates from an academic university hospital. All of the airlines that were listed in the World Airline Directory were contacted and asked to submit a sample of their documentation form for in-flight medical emergencies, provided that confidentiality would be maintained and the airlines' names would not be disclosed [7]. The documentation form data were evaluated independently by two authors (MS and SM) blind to the name and type of airline. The authors reviewed and classified all available data into five sub-categories: non-medical data, signs and symptoms, diagnosis, treatment and outcome. The collected items were compiled into an electronic database (Microsoft Excel for Windows, Microsoft Corp., Redmond, WA, USA). Furthermore, each documentation form was verified for its adherence to the sample "medical incident form" published by IATA in their Medical Manual [6].

## Results

A total of 1,318 airlines were contacted and invited to participate in this study (Table 1). A total of 10 airlines agreed to participate in the study. These airlines were based in Chile, Czechoslovakia, Germany, Switzerland, Turkey, the United Arab Emirates and the United Kingdom. Two hundred items were summarized into five sub-categories: non-medical data (63), signs and symptoms (68), diagnosis (26), treatment (22) and outcome (20).

**Table 1:** A total of 1,318 airlines were identified through the World Airline Directory

**Africa**	**121**
**Caribbean**	**42**
**Central America**	**15**
**Central Asia**	**38**
**Europe**	**399**
**Far East**	**73**
**Indian Subcontinent**	**58**
**Middle East**	**92**
**North America**	**205**
**Oceania**	**71**
**South America**	**99**
**South East Asia**	**105**

The two most frequent items in each sub-category were "date of incidence" and "passenger's name" (non-medical data), "description of the injury" and "pulse frequency/minute" (signs and symptoms), "convulsive seizures" and "burns" (diagnosis), "application of oxygen" and "resuscitation" (treatment) and "documentation of diversion" and "death of a patient" (outcome). The items "date of incidence" and "passenger's name" were the only items that were documented in all 10 documentation forms described in this study. For exact details, see Tables 2, 3, 4, 5, 6.

**Table 2:** Non-medical data*

Airline	A	B	C	D	E	F	G	H	I	J
Items										
Advice given by a physician/health-care professional (Y/N)	X				X					
Aircraft details (Type, No. of passengers)										X
Aircraft registration number								X		
Cabin activity										X
Cabin floor condition										X
Cabin lighting										X
Communication - ACARS used (Y/N)	X								X	
Communication - High frequency used (Y/N)	X									
Communication - MedLink used (Y/N)				X						
Communication - Satcom used (Y/N)	X									
Date of incident	X	X	X	X	X	X	X	X	X	X
Delay (Y/N)								X		
Departure airport	X	X	X					X		
Destination airport	X	X	X					X	X	X
Doctor on board call (Y/N)	X								X	
Duration of occurrence	X									
Emergency contact					X					
Flight factors										X
Flight number	X	X	X	X	X	X		X	X	X
Flight phase										X
General flight and weather conditions								X		
Ground medical control contact (Y/N)	X			X						
Ground medical control contact not successful (Y/N)	X									
Ground medical control contact successful (Y/N)	X									
Health-care professional assistance (Y/N)	X			X					X	X
Liability Information					X					
License number of the physician		X								
Location of incident								X		
Name of the flight purser	X	X	X							
Name, address, field of the assisting physician/health-care professional	X	X	X	X	X	X		X	X	X
Passenger's home address	X		X		X		X	X	X	X
Passenger's name	X	X	X	X	X	X	X	X	X	X
Passenger's age (years)					X				X	
Passenger's date of birth	X	X	X	X			X	X	X	X
Passenger's email address		X								
Passenger's frequent flyer status	X									
Passenger's nationality							X			
Passenger's passport number							X			
Passenger's seat number	X	X		X				X	X	X
Passenger's sex	X	X		X				X	X	
Passenger's signature accepting treatment		X					X			
Passenger's signature refusing treatment			X				X	X		
Passenger's ticket number								X		
Passenger's weight								X		
Passenger's home telephone number	X	X						X		X
Physician on board (Y/N)	X								X	
Physician compensation offered (Y/N)				X						
Physician's email address		X		X						
Physician's passport number					X					
Physician's telephone number		X		X						
Pilot name		X				X				
Pilot's personnel number		X								
Pilot's signature		X								
Port health authority advised (Y/N)				X						
Pregnancy (Y/N)	X	X		X						
Purser's personnel number	X	X								
Purser's signature		X	X							
Signature of physician/health-care professional		X	X					X		
Time of occurrence	X		X	X			X	X	X	X
Duration of treatment				X						
Type of flooring										X
Weather										X
Witness details (Name/Address/Nationality/Passport No.)							X			X

*Details from 10 airlines (A-J) on the documentation of on board in-flight medical emergencies, sub-category non-medical data.

**Table 3:** Signs and symptoms*

Airline	A	B	C	D	E	F	G	H	I	J
Items										
Blood pressure (mmHg)	X	X	X	X				X		
Body temperature (°C)	X	X	X	X	X			X		
Cardiac arrest observed (Y/N)				X						
Chest pain (Y/N)								X		
Choking (Y/N)								X	X	
Clinical course (free text)								X		
Consciousness - aggressive (Y/N)	X			X						
Consciousness - anxious (Y/N)	X			X	X					
Consciousness - awake and cooperative (Y/N)	X		X					X		
Consciousness - confused (Y/N)	X			X						
Consciousness - drowsy (Y/N)	X									
Consciousness - no reaction (Y/N)	X		X		X			X	X	
Consciousness - partially responsive (Y/N)			X					X		
Consciousness after resuscitation (Y/N)	X			X						
Coughing (Y/N)	X			X						
Description of the injury/event (free text)	X		X	X			X	X	X	X
Diarrhea (Y/N)	X		X	X	X			X		
Dyspnea (Y/N)			X					X		
General condition collapsible (Y/N)	X									
General condition weak (Y/N)	X									
Headache (Y/N)									X	
Hyperventilation (Y/N)			X					X		
Incontinence (Y/N)									X	
Last Meal/Fluid Intake (free text)									X	
Nausea (Y/N)	X			X	X				X	
Oxygen saturation		X								
Pain - intensity (light/moderate/severe)	X			X	X					
Pain - localization	X			X						
Pain - quality (sharp/spasmodic/persistent/episodic)	X			X						
Patient aggressive (Y/N)					X					
Patient confused (Y/N)					X					
Patient dizzy (Y/N)				X						
Patient drowsy (Y/N)					X					
Patient faint (Y/N)				X	X					
Patient intoxicated (Y/N)				X					X	
Patient unconscious (Y/N)				X						
Patient weak (Y/N)				X						
Patient history - allergies	X	X		X	X				X	
Patient history - known medical condition	X		X	X	X			X	X	
Patient history - medication taken	X		X	X	X				X	
Pulse - hand (Y/N)	X									
Pulse - neck (Y/N)	X									
Pulse - palpable (Y/N)										
Pulse - pulseless (Y/N)	X									
Respiration - arrest (Y/N)	X			X						
Respiration - cleaning of the respiratory tract (Y/N)			X					X		
Respiration - frequency (/min)	X		X	X	X			X	X	
Respiration - mouth-to-mouth ventilation (Y/N)			X					X		
Respiration - respiratory difficulty (Y/N)	X				X					
Respiration - ventilation (Y/N)								X		
Skin - cold (Y/N)	X		X	X				X		
Skin - cyanotic (Y/N)	X		X	X				X		
Skin - dry (Y/N)			X					X		
Skin - highly reddened (Y/N)			X					X		
Skin - moist (Y/N)	X			X	X			X	X	
Skin - normal (Y/N)			X					X		
Skin - pale (Y/N)	X		X	X	X			X		
Skin - patchy (Y/N)	X			X						
Skin - rash (Y/N)				X	X					
Skin - rash location				X						
Skin - warm (Y/N)	X		X	X				X		
Unconsciousness - duration (min)									X	
Vertigo (Y/N)	X									
Vomiting (Y/N)	X		X	X	X			X	X	
Wound location				X						
Wound severity (mild/moderate/severe)				X	X					

*Details from 10 airlines (A-J) on the documentation of on board in-flight medical emergencies, sub-category signs and symptoms.

**Table 4:** Diagnosis*

Airline	A	B	C	D	E	F	G	H	I	J
Items										
Acute abdomen (Y/N)								X		
Alcohol abuse (Y/N)								X		
Allergic reaction (Y/N)								X		
Asthmatic attack (Y/N)			X					X		
Bone fracture (Y/N)	X		X					X		
Bruising (Y/N)	X		X							X
Burn (Y/N)								X		X
Burn (Y/N)	X		X					X		X
Childbirth (Y/N)								X		
Circulatory collapse (Y/N)			X					X		
Convulsive seizure (Y/N)	X		X	X	X			X		
Cut (Y/N)	X									X
Febrile convulsion (Y/N)								X		
Foreign body (Y/N)	X									
Heart attack (Y/N)			X					X		
Hyperglycemia (Y/N)			X					X		
Hypertensive crisis (Y/N)			X					X		
Hypoglycemia (Y/N)			X					X		
Intoxication (Y/N)								X		
Mental illness (Y/N)								X		
Pseudocroup (Y/N)								X		
Scald (Y/N)								X		
Sprain (Y/N)	X									X
Strain (Y/N)										X
Stroke (Y/N)			X					X		
Vaginal bleeding (Y/N)								X		
Wound (Y/N)	X							X		X

*Details from 10 airlines (A-J) on the documentation of on board in-flight medical emergencies, sub-category diagnosis.

**Table 5:** Treatment*

Airline	A	B	C	D	E	F	G	H	I	J
Items										
Bandage (Y/N)				X					X	X
Defibrillator used	X			X				X	X	
Defibrillator used (number of times)	X		X						X	
ECG monitoring (Y/N)				X						
Endotracheal intubation				X					X	
Extra comments on defibrillation			X							
i.v. line established (Y/N)			X	X				X		
Improvement with oxygen (Y/N)	X									
Medical treatment applied (free text)				X	X				X	
Medication given on board (Y/N)	X	X		X	X					
Medication source (patient/medical kit/other passenger)	X									
Onboard medical equipment/medication used (free text)				X	X			X		
Oxygen applied (Y/N)	X		X	X	X			X	X	
Problems with the defibrillator			X							
Pulse palpable after resuscitation (Y/N)	X			X						
Resuscitation duration		X								
Resuscitation performed (Y/N)	X	X	X	X				X	X	
Splinting (Y/N)			X					X		
Time of defibrillation			X	X						
Total time of monitoring				X						
Ventilation after resuscitation (Y/N)	X			X						
Wound care (Y/N)								X		

*Details from 10 airlines (A-J) on the documentation of on board in-flight medical emergencies, sub-category treatment.

**Table 6:** Outcome*

Airline	A	B	C	D	E	F	G	H	I	J
Items										
Crew fit to operate (Y/N)	X									
Crew needs Critical Incident Stress Management program (CISM) (Y/N)	X									
Crew uses Critical Incident Stress Management program (CISM) (Y/N)	X									
Description of the outcome (free text)					X					
Diversion (Y/N)	X		X	X				X		
Diversion site			X							
Diversion time			X					X		
Further treatment - continuation of the flight (Y/N)								X		
Further treatment - in-patient (Y/N)								X		
Further treatment - none (Y/N)								X		
Further treatment - out-patient (Y/N)								X		
Health condition improved (Y/N)	X		X					X		
Health condition unchanged (Y/N)			X					X		
Health condition worsened (Y/N)			X					X		
Patient deboards with medical help (Y/N)				X						
Patient dies on board (Y/N)	X		X	X				X		
Patient leaves the aircraft on a stretcher (Y/N)	X			X						
Patient leaves the aircraft in a wheelchair (Y/N)	X			X						
Patient leaves the aircraft without help (Y/N)	X			X						
Patient recovered before landing (Y/N)				X						

*Details from 10 airlines (A-J) on the documentation of on board in-flight medical emergencies, sub-category outcome.

When compared to the sample "medical incident report form" published by the IATA, we found that no airline (0/10) included in this study adhered to the latter form.

## Discussion

This study is the first which investigates forms of documentation for in-flight medical emergencies that are currently provided in the emergency medical kits on board commercial airlines. Although we have to keep in mind that the results of this study are not necessarily representative due to selected samples, we were still able to observe a high degree of variance among the forms of documentation that are currently in use by the 10 different airlines in the present study. This is, however, not necessarily the case in other airlines which did not participate in our study.

If airline passengers become ill in the cabin during flight, the majority of airlines carry medical equipment, which enables physicians and medical professionals on board to treat the patient [8,9]. Previous studies have shown a help rate of 85% for all in-flight medical emergencies [4]. According to the recommendations made by the IATA, in-flight medical emergencies should be documented properly for a variety of reasons [6]. The standards and recommendations of the International Civil Aviation Organization (ICAO) also list an "incident record form" as a necessary part of the first aid kit [10]. Furthermore, other authors have also advised proper documentation for the passengers that offer medical assistance during in-flight medical emergencies [11]. The reason for this recommendation is obvious. Although doctors and medical professionals who offer assistance on board an aircraft are covered by the Good Samaritan law, basic documentation, including the patient's symptoms, diagnosis, treatment and administered medication and dosage, is essential in the event of a legal dispute [11]. Similar to any professional medical contact on the ground, documentation of an in-flight medical emergency is not only desirable from a medical standpoint but also from a legal point of view. Furthermore, paramedics and emergency physicians on the ground rely on important medical information and data, which can be secured in writing in-flight and facilitates the initial assessment of the course of an illness from its inception in the air to the point of patient handover. Most importantly, standardized documentation is necessary to facilitate data comparison in an international environment, such as in the aviation and aerospace industry, which includes over 2,000 different airlines worldwide. Each airline should develop its own medical care and incident policy. Without an incident reporting system, no incident management process can be developed. When faced with a patient who requires acute care under special circumstances, such as in flight in an airline cabin, such a process is highly desirable.

Because there is no central registry, some airlines analyze their own events. The majority of passenger transportation airlines, however, are not documenting medical emergencies on board their aircrafts [4]. Because evidence suggests epidemiologic research of in-flight medical emergencies has an important impact on updating recommendations for the contents of emergency medical kits, reassessment of the present situation is necessary [5]. Ruskin has provided strong arguments in favor of a centralized registry of in-flight medical emergencies and states that it would tremendously facilitate the epidemiologic research of in-flight medical emergencies and the development of training materials for physicians preparing to volunteer or assist physicians in evaluating the patients' fitness to fly [5]. Furthermore, a registry would enable the airlines to provide information about the true incidence of specific illnesses that occur during flight because data would not be based on any one single airline.

Based on the results of this study, we believe that it is necessary for the appropriate national and international authorities and organizations to discuss a standardized form of documentation for in-flight medical emergencies. Based on the current data of in-flight medical emergencies and the finding that documentation varies greatly, it is impossible to initiate larger studies with multiple airlines from different regions of the world. The aeromedical community today is dependent on small studies with one or two participating airlines, which document by chance similar items that can be analyzed together. The scientific impact of these studies, however, is low due to limited data quality. Therefore, in the future, we should discuss standardized documentation protocols or an international registry for in-flight medical emergencies.

There are limitations that need to be acknowledged and addressed regarding the present study. The generalizability of the research findings are limited because this study includes only a very small number of participating airlines and there is a high degree of variance due to selected samples.

## Conclusion

The data in this study illustrate a large variation in the documentation of in-flight medical emergencies by 10 different airlines. A higher degree of standardization is preferable to facilitate aeromedical research and to meet the recommendations that are published by the IATA.

## Key messages

• Documentation of in-flight medical emergencies is inadequate due to large variation

• A standardized form of documentation for in-flight medical emergencies is desirable

• A central registry for in-flight medical emergencies will help to facilitate aeromedical research

## Abbreviations

ACARS: aircraft communications addressing and reporting system; CISM: Critical Incident Stress Management program; EU: European Union; IATA: International Air Transport Association; ICAO: Civil Aviation Organization; mmHg: millimetres of mercury.

## Competing interests

All authors hereby disclose any commercial associations that may pose or create a conflict of interest with the information presented in this manuscript. The authors report no conflicts of interest. The authors alone are responsible for the content and writing of the paper.

## Authors' contributions

MS participated in the study design, data analysis and interpretation of the data as well as the writing of the manuscript. SM participated in the data analysis. DS participated in the data analysis, literature search, revision of the bibliography, the revision and editing of most of the manuscript. PA participated in the data analysis of the manuscript. FGB participated in the data analysis, the revision and editing of part of the manuscript. MS, SM, DS, PA and FGB critically revised the manuscript for intellectual content. All authors read and approved the final manuscript.
